# Application of non-invasive transcranial photobiomodulation in ischemic stroke: Mechanisms and current insights

**DOI:** 10.1016/j.isci.2025.114254

**Published:** 2025-11-27

**Authors:** Lifeng Tang, Xiaohan Li, Jiliang Kang, Yuli Huang, Youliang Wen, Min Tang

**Affiliations:** 1 Neurological Rehabilitation Department, Ningbo Rehabilitation Hospital, Ningbo, China; 2 Faculty of Rehabilitation, Gannan Medical University, Ganzhou, China

**Keywords:** Cardiovascular medicine, Health sciences, Medicine, Neurology

## Abstract

Ischemic stroke (IS), comprising 65.3% of the 11.9 million new stroke cases worldwide in 2021, is a leading cause of disability and mortality due to cerebral vascular occlusion and subsequent ischemia. The increasing prevalence, particularly in aging populations such as China, underscores the urgent need for novel therapeutic strategies. Conventional treatments, including thrombolysis and surgery, are constrained by narrow therapeutic windows and risks such as hemorrhage. Transcranial photobiomodulation (tPBM), a non-invasive technique utilizing red or near-infrared light (630–1300 nm), has emerged as a promising intervention for IS. This review synthesizes the pathological features of IS, including blood-brain barrier disruption, oxidative stress, and neuroinflammation, and elucidates the molecular mechanisms of tPBM, such as enhanced mitochondrial function, increased cerebral blood flow, and upregulation of neurotrophic factors. Preclinical and clinical evidence demonstrate tPBM’s potential to mitigate neuronal damage and promote recovery across acute, subacute, and chronic phases of IS. By evaluating current applications, this study aims to provide a theoretical foundation for optimizing treatment parameters, enhancing clinical outcomes, and guiding future rehabilitation strategies for patients with stroke.

## Graphical abstract

**Figure:**
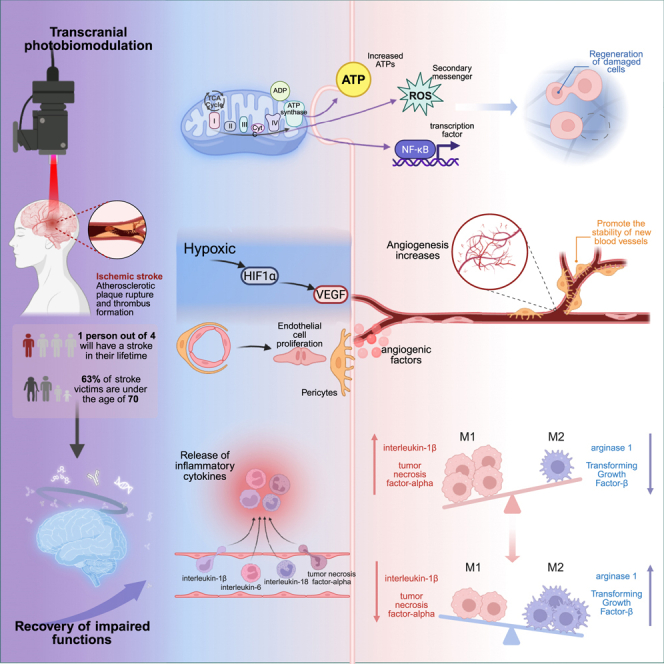

## Introduction

Stroke, an acute cerebrovascular event, is classified into ischemic and hemorrhagic subtypes. According to the Global Burden of Disease Study 2021, approximately 11.9 million new stroke cases were reported globally in 2021, with ischemic stroke (IS) accounting for 65.3%.^1^ IS results from cerebral vascular occlusion, often by thrombi formed locally or emboli from distant sites, such as the heart, leading to regional ischemia and hypoxia. In China, over one million new stroke cases occur annually, with the rising aging population exacerbating the socioeconomic burden.^2^ Acute IS manifests as hemiparesis, aphasia, or visual deficits, primarily affecting brain regions with disrupted blood supply. Persistent motor, linguistic, and cognitive impairments often impair patients’ quality of life.^3^ Current treatments, including thrombolysis, anticoagulation, blood pressure management, and surgical interventions, aim to restore blood flow and minimize tissue damage. However, these approaches are limited by narrow therapeutic windows and risks such as hemorrhage.^4^ Consequently, novel, non-invasive interventions are urgently needed to address these challenges. Transcranial photobiomodulation (tPBM), also known as low-level light therapy, is an emerging non-invasive neurotherapeutic technique that employs red or near-infrared (NIR) light (620–1440 nm, with an optimal therapeutic window of 600–900 nm) to stimulate cellular repair and neuroprotection.^5^ By delivering low-intensity light to the scalp, tPBM targets brain tissue to promote neural recovery, reduce inflammation, and enhance cerebral blood flow.^6^ Preclinical and early clinical data show that tPBM therapy can extend the treatment window to 24–72 h post-stroke and even the subacute phase,^7^ maintaining neuroprotection, reducing infarct volume, and promoting long-term neurological recovery without increasing adverse risks such as bleeding. Preclinical studies have demonstrated its efficacy in various neurological conditions, including stroke,^8^ traumatic brain injury,^9^ Alzheimer’s disease,^10^ Parkinson’s disease,^11^ and depression.^12^ Although most evidence stems from animal models, tPBM shows promise in mitigating oxidative stress, inflammation, and neuronal loss while promoting functional recovery and systemic benefits.^13^ This review examines the pathological mechanisms of IS, the neurophysiological effects of tPBM, and its clinical applications, aiming to provide a theoretical basis for optimizing treatment protocols and advancing novel rehabilitation strategies.

## Pathological features and mechanisms of ischemic stroke

**Figure 1:**
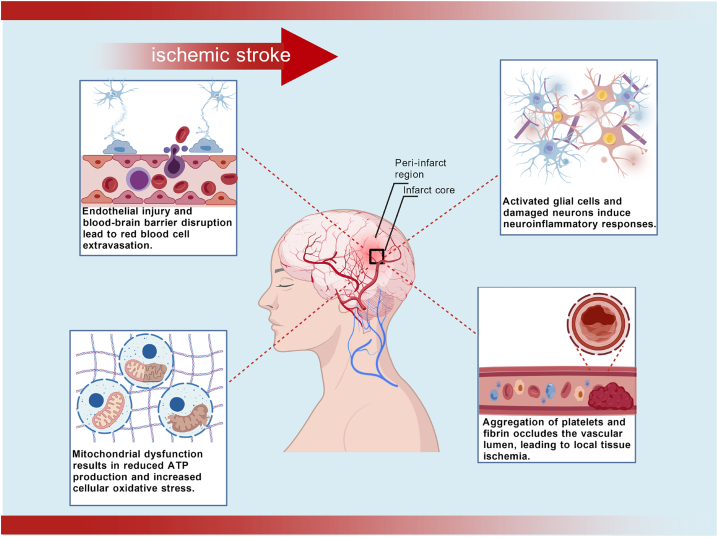
The pathological cascade of ischemic stroke Vascular endothelial damage and blood-brain barrier disruption induce red blood cell extravasation; mitochondrial dysfunction reduces adenosine triphosphate synthesis and amplifies oxidative stress; activated glial cells and damaged neurons initiate neuroinflammation; platelet-fibrin aggregates obstruct the vascular lumen, causing local ischemia; and an irreversible infarct core forms with a salvageable penumbra. This process disrupts core networks, such as the default mode network, impairing cognitive and motor recovery. These interacting mechanisms collectively drive neuronal death and brain functional impairment.

Is triggers a complex and dynamic pathological cascade, primarily involving the dysfunction of the neurovascular unit (NVU), which encompasses neurons, endothelial cells, and supporting structures. The hallmark of IS is reduced cerebral perfusion due to vascular occlusion, initiating a series of cellular responses, including blood-brain barrier (BBB) disruption, oxidative stress, inflammation, and neuronal network dysfunction (Figure 1).^14^

### Damage to cerebral vascular endothelial cells

Cerebral ischemia initially impairs the function of cerebral vascular endothelial cells, reducing their ability to regulate cerebral blood flow. The activation of coagulation mechanisms and subsequent acute vascular occlusion rapidly trigger perivascular inflammation while markedly increasing BBB permeability. This facilitates peripheral immune cell infiltration and the entry of harmful molecules, exacerbating brain tissue damage.^15^ Oxidative stress products, such as reactive oxygen species (ROS) and proteolytic enzymes such as matrix metalloproteinases (MMPs), directly disrupt BBB integrity and induce cerebral edema.^16^ Under ischemic conditions, enzymes including NADPH oxidase, xanthine oxidase, and nitric oxide synthase (NOS) undergo abnormal uncoupling, generating superoxide while reducing vasoprotective nitric oxide (NO).^17^ This dual effect weakens endothelial cells’ capacity to maintain microcirculation and intensifies vascular damage via oxidative stress. The decline in NO, along with the loss of its anti-inflammatory, anti-adhesive, and anti-aggregatory properties, promotes platelet aggregation and leukocyte adhesion, further impairing microvascular flow regulation—a critical factor in early vascular inflammation in IS.^18^ Additionally, within 1 h of ischemia, pericytes detach from the basement membrane, migrate to hypoperfused regions, and overexpress regulator of G-Protein Signaling 5 (RGS5) while secreting large amounts of MMP-9 upon hyperactivation,^19^ further compromising the structural integrity of the BBB and NVU.

### Mitochondrial damage and oxidative stress

IS acute cerebral ischemia and hypoxia elicit complex cellular stress responses, with mitochondria—the central hub of energy metabolism—being the first to sustain damage, playing a pivotal role in brain injury progression. Ischemia disrupts the mitochondrial electron transport chain, reducing adenosine triphosphate (ATP) synthesis and shifting cells toward anaerobic metabolism, leading to lactate accumulation and acidosis that destabilize cellular homeostasis.^20^ Concurrently, mitochondrial dysfunction amplifies ROS production, overwhelming endogenous antioxidant systems such as glutathione (GSH) and causing lipid peroxidation, protein modification, and DNA fragmentation.^21^ ROS also triggers the opening of the mitochondrial permeability transition pore (mPTP), destabilizing membrane potential, inducing mitochondrial swelling, and releasing cytochrome *c* (Cyt-C) to activate the caspase pathway and programmed cell death.^22^ Studies in cerebral ischemia models reveal that the oxidative activation of mitochondrial fission proteins such as Drp1 promotes excessive mitochondrial fragmentation, amplifying ROS accumulation and forming a positive feedback loop of oxidative stress.^23^ Additionally, oxidative stress suppresses the expression of mitochondrial fusion proteins (e.g., Mfn2 and Opa1), disrupting mitochondrial dynamics and sustaining apoptotic pathways.^24^ Bioinformatic analyses further indicate that key genes in mitochondrial ROS and GSH pathways exhibit significant differential expression in stroke samples, correlating closely with immune cell infiltration and inflammatory factor expression, suggesting that oxidative stress may aggravate brain tissue damage by modulating the immune microenvironment.^25^

### Decline in whole-brain functional connectivity

The pathological changes in IS extend beyond the local lesion, disrupting widespread brain functional networks. The coupling between structural connectivity (SC) and functional connectivity (FC) diminishes,^26^ with mechanisms tied to the disruption and imbalanced reorganization of intrinsic brain networks, including the default mode network (DMN), salience network (SN), and central executive network (CEN).^27^ Evidence from diffusion tensor imaging (DTI) and resting-state functional magnetic resonance imaging (rs-fMRI) demonstrates that ischemic lesions impair local white matter fiber integrity (e.g., reduced fractional anisotropy), hindering dynamic interactions between networks, particularly affecting anatomical connectivity in circuits such as the basal ganglia-frontal lobe loop.^28^ Concurrently, FC networks exhibit pronounced abnormalities, with reduced cohesiveness in the DMN and CEN and increased compensatory hyperconnectivity in distant brain regions. This FC disruption, combined with SC damage, results in decreased SC-FC coupling strength.^29^ Mechanistically, this reduction reflects both the direct weakening of FC due to white matter nerve fiber disruption in the lesion area and impaired information transmission efficiency within intrinsic brain networks.

## Neurophysiological effects of transcranial photobiomodulation in ischemic stroke

tPBM leverages low-intensity red or NIR light to modulate neural function, primarily by targeting cytochrome *c* oxidase (CCO), a key mitochondrial chromophore. Light absorption by CCO initiates a cascade of neurophysiological effects, addressing multiple aspects of IS pathology, including energy deficits, inflammation, and neuronal loss. tPBM enhances mitochondrial oxidative phosphorylation, increases ATP synthesis, and reduces oxidative stress while promoting cerebral blood flow (CBF), iymphatic drainage and clearance, anti-inflammatory responses, and neurotrophic factor expression. These effects create a favorable microenvironment for neuroplasticity, including neurogenesis, synaptogenesis, and angiogenesis, across acute, subacute, and chronic phases of IS.

### Mitochondrial cytochrome *c* oxidase and energy metabolism

CCO, the terminal enzyme of the mitochondrial electron transport chain (ETC) (also known as complex IV), catalyzes the reduction of oxygen to water. Its structure comprises two heme centers (a and a_3_) and two copper centers (CuA and CuB), enabling photon absorption at specific wavelengths, notably in the red (600–700 nm) and NIR light (760–940 nm) spectra.^30^ In the acute phase of IS, hypoxia and metabolic suppression enhance the binding of endogenous NO to CCO, reversibly inhibiting its activity. This inhibition disrupts electron transport efficiency, causing mitochondrial membrane potential (MMP) imbalance, impaired oxidative phosphorylation, and reduced ATP production. Consequently, these alterations induce intracellular metabolic disturbances, calcium overload, and ROS bursts, promoting apoptosis. Thus, CCO serves as a critical target for tPBM to mitigate neuronal damage in IS. tPBM targets CCO’s chromophore centers to trigger a photosensitive response.^31^ Photon absorption induces photodissociation, releasing inhibitory NO,^32^ thereby restoring electron transport chain function and mitochondrial oxygen reduction. This is accompanied by MMP repolarization, increased oxygen consumption rate (OCR), and enhanced aerobic glucose metabolism,^33^ ultimately improving ATP synthesis and alleviating energy deficits in IS.^34^

**Figure 2:**
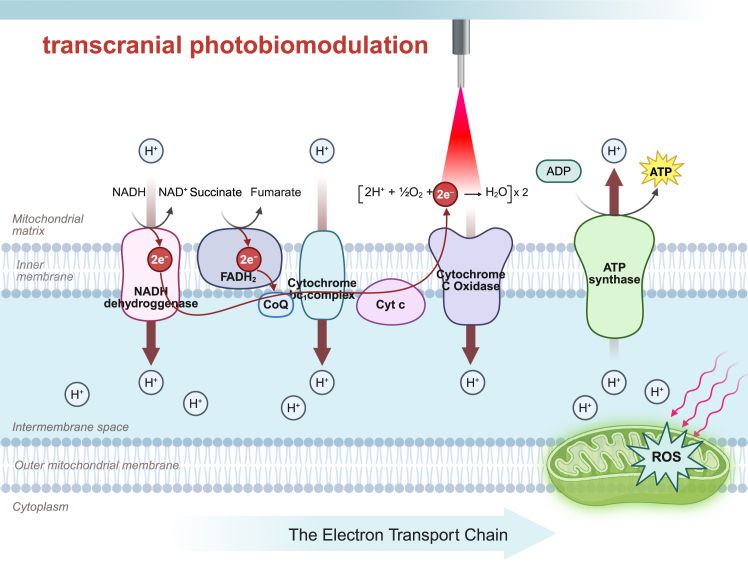
Transcranial photobiomodulation exerts its effect through the electron transport chain of mitochondria Transcranial photobiomodulation (tPBM) enhances the activity of cytochrome *c* oxidase (CCO) in the mitochondrial electron transport chain (ETC), promoting electron transfer and proton gradient formation across the inner membrane. This leads to increased ATP production via ATP synthase and regulated generation of ROS. These events elevate neuronal respiration and cellular metabolic capacity, forming the basis of tPBM’s neuroprotective effects.

Additionally, specific tPBM wavelengths amplify CCO enzymatic activity.^35^ Beyond this, tPBM activates retrograde mitochondrial signaling, where light-stimulated mitochondria signal the nucleus to regulate gene expression, enhancing mitochondrial function and biogenesis.^36^ A separate, non-CCO-dependent mechanism involves the interfacial water layer (IWL) near the mitochondrial inner membrane. Under oxidative stress, increased IWL viscosity hinders ATP synthase γ-subunit rotation and ATP production.^37^ tPBM at 670 nm reduces IWL viscosity via light-water interactions, restoring ATP synthase activity.^38^ Together, these mechanisms, alongside CCO photoactivation, collectively enhance mitochondrial energy metabolism in IS (Figure 2).

### Anti-inflammatory and antioxidant effects

The anti-inflammatory and antioxidant effects of tPBM IS treatment primarily rely on its regulation of intracellular ROS levels and inflammatory signaling pathways.^39^ tPBM activates CCO in mitochondria through photon stimulation, inducing transient, low-level ROS production, termed “oxidative stress.” This moderate oxidative stress acts as a signaling molecule, activating downstream cytoprotective pathways, including the suppression of cyclooxygenase-2 (COX-2), modulation of apoptosis, and downregulation of nuclear factor-kappa B (NF-κB) transcription pathways,^40^ thereby maintaining redox homeostasis.^41^ However, high energy densities tPBM (e.g., 50 J/cm^2^) may trigger excessive cytoplasmic ROS accumulation,^42^ activating the ROS-induced ROS release (RIRR) mechanism in adjacent mitochondria, forming a destructive feedback loop that exacerbates local cellular damage.^43^

Additionally, IS is typically accompanied by the release of proinflammatory cytokines, such as interleukin-1β (IL-1β), IL-6, tumor necrosis factor-alpha (TNF-α), and IL-18,^44^ with neuroinflammation being a key pathological contributor to neuronal loss and cognitive impairment. Studies demonstrate that tPBM inhibits reactive activation of astrocytes and microglia, reducing the expression of the AIM2 inflammasome and the microglial marker ionized calcium-binding adapter molecule 1 (Iba1),^45^ thereby decreasing inflammatory cell infiltration. Furthermore, tPBM promotes microglial polarization from the proinflammatory M1 phenotype (marked by cluster of differentiation 86 [CD86] and inducible nitric oxide synthase [iNOS]) to the anti-inflammatory M2 phenotype (marked by arginase 1 [ARG1] and cluster of differentiation 206 [CD206]),^46^ facilitating clearance of proinflammatory cytokines in the brain. Moreover, tPBM exerts anti-inflammatory effects by enhancing lymphatic flow in meningeal lymphatic vessels (MLVs).^47^ Low energy densities tPBM induce mesenteric lymphatic vessel relaxation and reduce contraction amplitude,^48^ effectively alleviating chronic inflammation in brain tissue and promoting tissue repair.

### Neurotrophic factors and synaptic plasticity mechanisms

tPBM plays a significant role in neural repair following IS by modulating neurotrophic factor expression and enhancing synaptic plasticity, ultimately promoting neurogenesis. Brain-derived neurotrophic factor (BDNF) serves as a central mediator of tPBM’s therapeutic effects. Studies show that 632.8 nm light activates intracellular IP3 receptors, increasing Ca^2+^ influx and stimulating the ERK/CREB signaling pathway, which boosts BDNF mRNA and protein synthesis.^49^ This process enhances dendritic morphology and synaptic transmission. BDNF also regulates synaptophysin (SYP) upstream, promoting axonal growth and stabilizing synaptic connections, thus facilitating neural network reconstruction.^50^ Research by Xuan et al. demonstrates that 810 nm irradiation over 1–3 days increases dendritic branching, fiber connectivity, and SYP expression, underscoring this pathway’s role in synaptic remodeling.^51^

Additionally, 660–670 nm light elevates BDNF levels in the hippocampus and cortex while suppressing oxidative stress-induced apoptosis, mitigating neuronal atrophy and synaptic loss caused by ischemia.^52^^,^^53^ Moreover, Feng et al. found that tPBM reverses the downregulation of postsynaptic spinophilin and presynaptic SYP induced by oxygen-glucose deprivation (OGD) in IS models, providing a molecular foundation for synaptic structural and functional recovery.^54^ Beyond plasticity, tPBM activates dormant neural stem cells (NSCs) and promotes the proliferation, migration, and differentiation of neural progenitor cells (NPCs), enhancing endogenous regeneration in neurogenic regions such as the subventricular zone (SVZ) and hippocampal dentate gyrus (DG).^51^ These combined effects create a supportive microenvironment for brain tissue regeneration and neural network remodeling.

### Mechanisms of improved cerebral blood flow and oxygenation

In IS models, inadequate cerebral perfusion due to ischemia is a key pathological feature, and tPBM offers a novel therapeutic approach to address this. tPBM stimulates soluble guanylate cyclase (sGC) to produce cyclic guanosine monophosphate (cGMP), activating protein kinase G (PKG). This cascade promotes Ca^2+^ reuptake and opens calcium-activated potassium channels, reducing intracellular Ca^2+^ levels, inhibiting myosin light-chain kinase phosphorylation, and inducing vascular smooth muscle relaxation, thereby enhancing CBF.^40^ NO, a critical vasodilator, is also modulated by tPBM. It activates CCO, releasing bound NO, which reduces intracellular Ca^2+^ via the cGMP–PKG pathway, further improving perfusion and oxygenation.^55^ tPBM also enhances the phosphorylation of endothelial nitric oxide synthase (eNOS) at serine 1177, a process dependent on wavelength and intensity.^56^ For instance, 590 nm LED increases CCO/NO activity and NO release,^57^ while 808 nm NIR light over 45 min elevates cortical CBF and NO levels, an effect reliant on intact eNOS function.^58^ Kashiwagi et al. showed that 1064 nm tPBM preconditioning improves CBF, reduces infarct volume, and enhances neurological outcomes in ischemia-reperfusion models—an effect absent in eNOS phosphorylation-deficient mice, highlighting eNOS’s pivotal role.^8^^,^^59^

Additionally, tPBM improves erythrocyte function and oxygen delivery at the microcirculatory level. Though erythrocytes lack mitochondria, tPBM excites electrons in hemoglobin’s porphyrin ring, stabilizing Fe^2+^ coordination and enhancing oxygen-carrying capacity.^60^ Studies using 850 nm light report increased oxyhemoglobin levels, indicating improved local oxygen supply.^61^ tPBM also modulates erythrocyte membrane proteins, such as aquaporin-1,^62^ to enhance deformability, and activates Na^+^/K^+^-ATPase to stabilize membrane charge and ion transport, reducing aggregation and optimizing hemorheology.^63^ These effects collectively underscore tPBM’s potential to improve oxygenation and maintain microcirculatory stability in IS.

### Promotion of vascular remodeling

In brain injury driven by ischemia and hypoxia, targeted angiogenesis strategies are increasingly valued for their therapeutic potential. tPBM promotes angiogenesis by upregulating the expression of hypoxia-inducible factor 1α (HIF-1α) and vascular endothelial growth factor (VEGF) expression^64^ while suppressing matrix metalloproteinase-2 (MMP-2) activity,^65^ thus enhancing the ischemic microenvironment and fostering neovascularization. Cury et al.^66^ showed in an animal model that irradiation at 780 nm with 40 J/cm^2^ energy density effectively induces these molecular changes. Salgado et al.^67^ reported that tPBM increases systolic and diastolic blood flow velocities in the middle cerebral artery (MCA) and basilar artery (BA) of elderly women, alongside reduced pulsatility and resistance indices, suggesting improved cerebrovascular compliance. Furthermore, tPBM mobilizes type 2 pericytes, aiding blood flow reconstruction and capillary formation in ischemic areas.^68^ Under ischemic-hypoxic conditions, pericytes detach from the endothelium, secrete angiogenic factors to drive endothelial migration and proliferation, and later reattach to nascent vessels, supporting vascular maturation and stability.^69^ These findings elucidate tPBM’s mechanistic role in post-stroke microcirculatory repair.

### Effects of photobiomodulation on gated channels

In addition to its classical mitochondrial and cerebrovascular effects, recent studies suggest that the modulation of Transient Receptor Potential (TRP) channels may represent a novel mechanistic pathway of tPBM These non-selective cation channels are essential for sensing temperature, mechanical force, and chemical stimuli, and their ability to regulate intracellular calcium signaling makes them a key therapeutic target in neurological diseases.^70^ Within this family, the TRPV1 channel is particularly pivotal in the context of IS.^71^ Calcium overload, a hallmark of IS neuronal damage, is mediated by TRPV1’s high Ca^2+^ permeability and its role in glutamate excitotoxicity.^72^ Pharmacological studies have underscored the therapeutic potential of this target: in middle cerebral artery occlusion (MCAO)/reperfusion models, modulation of TRPV1 indirectly reduced glutamate-induced calcium influx by downregulating N-Methyl-D-Aspartate (NMDA) receptor subunits GluN1 and GluN2B.^73^ An effect that is absent in TRPV1 knockout mice, thus confirming the viability of TRPV1 as a target.^72^ Importantly, recent findings demonstrate that tPBM can directly modulate TRPV1 activity, thereby altering calcium flux and attenuating excitotoxic stress.^74^ Zupin et al. showed that 970 nm tPBM downregulates TRPV1 expression in human sensory neuron models. In their experiments, capsaicin was used as a tool to activate the TRPV1 channel, and they found that tPBM-treated cells exhibited significant resistance to this capsaicin-induced calcium influx. This indicates that tPBM can suppress excitotoxicity by reducing the cellular calcium load.^75^ Similarly, in 50B11 rat sensory neurons, tPBM pretreatment inhibits TRPV1-mediated rapid calcium influx.^76^

**Figure 3:**
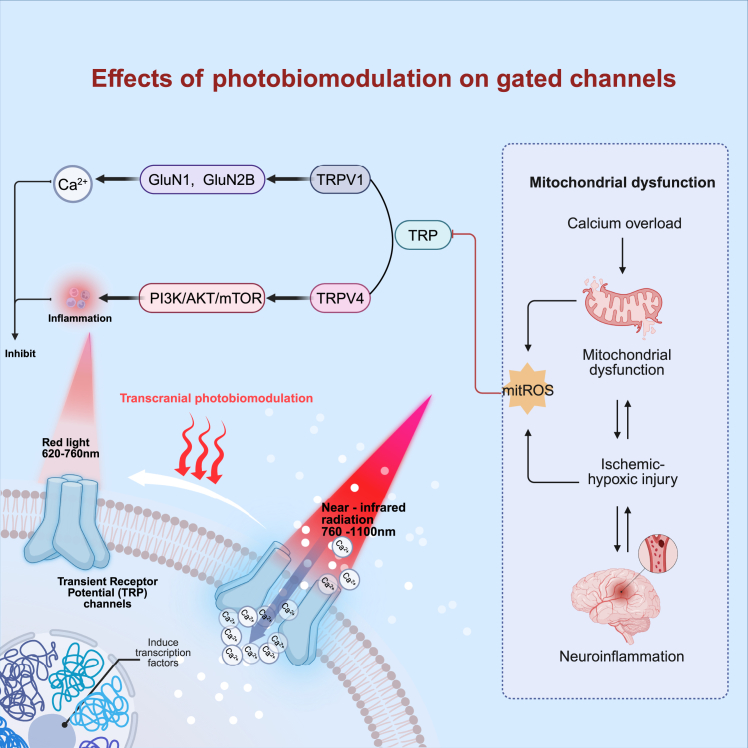
Transcranial photobiomodulation mediated regulation of transient receptor potential channels in ischemic neuroprotection Transcranial photobiomodulation (tPBM) regulates transient receptor potential (TRP) channels such as TRPV1 and TRPV4, reducing pathological calcium influx, mitochondrial dysfunction, and neuroinflammation. This mechanism complements classical mitochondrial and vascular pathways of tPBM in ischemic neuroprotection.

Furthermore, the modulatory effect of tPBM extends to other calcium-permeable TRP channels, such as TRPV4. Under ischemic conditions, TRPV4 can be activated by mitochondrial reactive oxygen species (mitROS), which in turn drives pathological Ca^2+^ influx, induces mitochondrial dysfunction, and exacerbates neuronal damage.^77^ The inhibitory effect of tPBM on TRPV4 has been validated in synovial cell models, where Meng et al. found that 630 nm red light suppresses TRPV4 activity and its downstream PI3K/AKT/mTOR signaling pathway, thereby modulating inflammation and promoting cell survival.^78^ Collectively, tPBM exerts neuroprotective effects in IS by regulating TRP channel activity and calcium flux to maintain intracellular homeostasis under ischemic-hypoxic conditions (Figure 3).

### Effects on intrinsic brain networks

In addition to the direct effects on mitochondrial function, cerebral blood circulation, and inflammatory responses, tPBM modulates FC between brain regions, facilitating topological reorganization and functional recovery of brain networks following injury. In IS, core networks such as the DMN, SN, and CEN exhibit imbalances due to disrupted anatomical structures and functional coordination. tPBM offers potential as a novel intervention for neural rehabilitation by enhancing the intrinsic states of these networks across multiple dimensions. Naeser et al. reported that six weeks of tPBM treatment significantly strengthens FC within the SN and CEN, with improvements linked to enhanced executive function, attention, language, and mood.^79^ Magnetic resonance spectroscopy further revealed elevated N-acetylaspartate (NAA) levels in the anterior cingulate cortex (ACC), indicating that tPBM boosts local metabolic capacity and supports neural network reconstruction. Additionally, tPBM reduces aberrant connectivity dispersion among key networks, refining functional pathways. For example, Chao et al. observed sharper connectivity between the ACC and anterior insula—both SN components—post-tPBM, suggesting more efficient attention allocation.^80^ This may reflect diminished reliance on compensatory mechanisms and more effective neural reorganization.

Mechanistically, tPBM’s neuromodulatory effects appear tied to the anatomical alignment between stimulated regions and target networks, potentially enhancing function by increasing metabolic activity in specific nodes, such as the prefrontal cortex within the CEN.^81^ Studies on stroke-related aphasia further demonstrate that targeting language-related CEN nodes yields notable therapeutic outcomes.^82^ Dmochowski et al., using functional magnetic resonance imaging (fMRI), found a 15% increase in FC between stimulated regions and multiple brain areas, particularly in the stimulated hemisphere, highlighting tPBM’s dual role in local modulation and broader network integration.^83^ However, El Khoury et al. detected no significant changes in four resting-state networks pre- and post-tPBM, suggesting that its effects may manifest only when brain circuit function is altered, such as during neuronal imbalance or disrupted homeostasis.^84^ Thus, under pathological conditions such as IS, tPBM’s capacity for network repair merits further exploration.

### Promote lymphatic drainage and clearance

**Figure 4:**
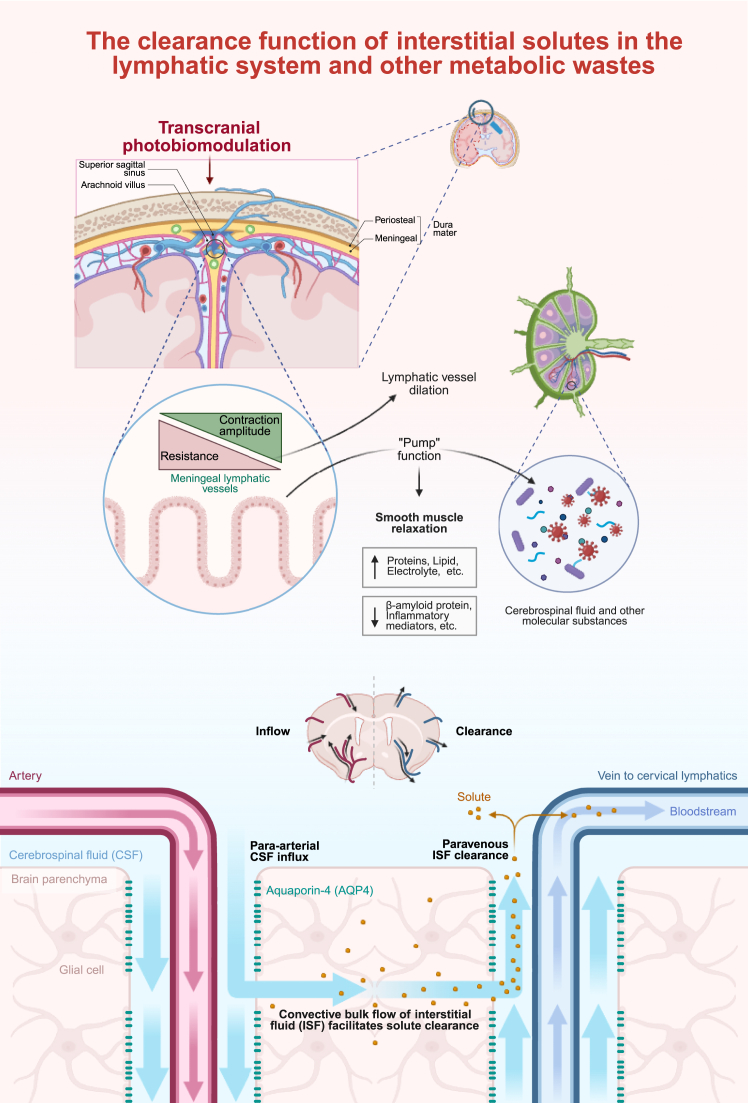
Transcranial photobiomodulation mediated modulation of lymphatic and glymphatic systems for cerebral clearance Transcranial photobiomodulation (tPBM) enhances cerebrospinal and interstitial fluid exchange by promoting aquaporin-4 (AQP4) polarization and meningeal lymphatic activity. These effects facilitate the removal of metabolic waste and support cerebral homeostasis.

Recent studies have found that tPBM also plays a unique role in stimulating meningeal lymphatic vessels and enhancing cerebral waste clearance.^48^ While traditionally attributed to CCO, wavelengths around 1200 nm may involve an alternative mechanism. This band coincides with the absorption peak of water molecules, enabling deeper penetration through the skull and brain tissue, where the energy is absorbed by “structured water layers” (also known as interfacial water).^85^ Light absorption by these layers induces charge separation,^86^ which resembles local pH shifts and may influence protein conformation,^87^ including the expression and polarization of aquaporin-4 (AQP4)—a circadian-regulated channel essential for cerebrospinal fluid (CSF) influx into the brain parenchyma.^88^ Emerging evidence suggests that tPBM promotes AQP4 polarization onto astrocytic endfeet and stimulates meningeal lymphatic vessels, optimizing CSF-Interstitial fluid (ISF) exchange to stabilize dysfunctional circadian fluctuations in influx and thereby enhancing glymphatic drainage efficiency in preclinical models^89^ (Figure 4).

Additionally, preclinical studies across multiple brain injury models have further demonstrated tPBM’s capacity to strengthen lymphatic functionality and accelerate the clearance of diverse pathological substrates. For example, Li et al. demonstrated that tPBM accelerates lymphatic clearance of red blood cells after intraventricular hemorrhage by enhancing MLVs' function via NO-dependent mechanisms, leading to reduced hematoma volume and improved neurological recovery.^90^ In studies of aging and neurodegenerative models, tPBM has likewise been shown to restore the mitochondrial metabolism of lymphatic endothelial cells and reinforce the structural integrity of meningeal lymphatic vessels, thereby facilitating the clearance of amyloid-beta and advanced glycation end products and mitigating neuroinflammation and cognitive decline.^91^^,^^92^ Meanwhile, tPBM has also been shown to promote the lymphatic removal of inflammatory cytokines in D-galactose-induced mice,^93^ suggesting broader immunomodulatory benefits. Mechanistically, Semyachkina-Glushkovskaya et al. reported that tPBM at 1267 nm with low energy density (<10 J/cm^2^) induces singlet-oxygen generation within biological tissues, which in turn triggers lymphatic vessel relaxation and facilitates the transport of macromolecules. This supports the enhanced clearance of pathological substrates reported in the aforementioned studies.^94^

Together, these findings suggest that tPBM preserves the cerebral microenvironment by promoting coordinated brain-waste-clearance mechanisms. Moreover, tPBM appears to regulate immune cell migration, decrease trans endothelial resistance, and downregulate vascular endothelial cadherin (VE-cadherin),^95^ ultimately contributing to improved metabolic homeostasis. This enhanced clearance capacity holds particular relevance for IS, where accelerated removal of post-stroke metabolites, inflammatory cytokines, and hemoglobin degradation products may help mitigate cerebral edema and secondary neuroinflammation.^90^^,^^96^ While current evidence remains largely preclinical, the consistent efficacy observed across diverse pathological models underscores the translational potential of tPBM. Future studies should further assess the feasibility of applying tPBM to stimulate meningeal lymphatic function in IS, with particular attention to optimizing illumination timing and circadian-dependent AQP4 dynamics. Such strategies may provide an innovative avenue for early neuroprotection in IS.

## Impact of photobiomodulation parameters on therapeutic outcomes

**Figure 5:**
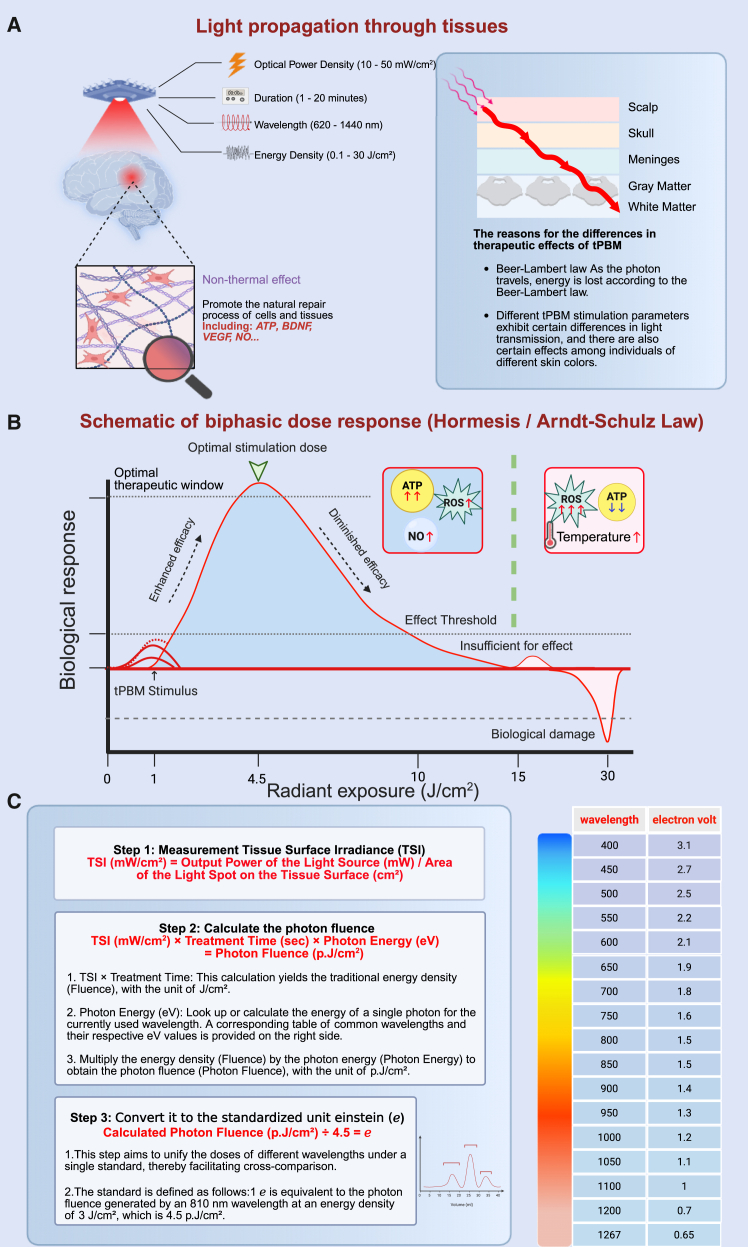
Optimizing transcranial photobiomodulation efficacy: parameters, biphasic response, and dose standardization critical parameters influencing transcranial photobiomodulation (tPBM) efficacy, alongside principles for optimizing therapeutic outcomes (A)This panel details key parameters for tPBM, including wavelength, optical power density, and energy density. It highlights how these, along with patient-specific factors such as skin tone, influence light penetration and therapeutic outcomes. Understanding these variables is crucial for effective tPBM treatment protocol design. (B)This panel illustrates the inverted U-shaped dose-response curve, also known as hormesis or the Arndt-Schulz Law. Optimal biological effects, such as enhanced ATP synthesis and anti-inflammatory activity, are observed within a defined “therapeutic window.” In contrast, doses that are too low are ineffective, and excessively high doses can lead to inhibitory/toxic effects or mitochondrial damage. (C)This panel outlines a three-step process for standardizing tPBM dose reporting. It explains the calculation of photon fluence and its conversion into the standardized einstein (ℯ) unit, which aids in reconciling dose conflicts across different studies. This standardization helps to improve cross-study comparability and reduce ambiguity in reported efficacies.

The efficacy of tPBM depends on several key parameters, summarized in Table 1. First, Wavelength (λ, in nm) is a primary determinant of tissue penetration; this is because the NIR light “therapeutic window” is a spectral range where major endogenous chromophores, such as water and melanin, exhibit low absorption—a property that permits deeper penetration of light into tissue.^97^ Second, Optical power density (OPd, in mW/cm^2^), which defines the energy delivery rate, is a critical parameter for balancing the desired therapeutic effect against thermal risk.^98^ Third, Energy Density (fluence, in J/cm^2^) quantifies the total dose per unit area and directly dictates the magnitude and nature of the biological effect.^70^ Fourth, patient-specific factors such as skin tone (melanin content) also affect light absorption and scattering, potentially altering the final intracranial fluence.^99^ Collectively, these interacting variables highlight the complexity of tPBM dosing. Therefore, a comprehensive strategy is essential when designing treatment protocols (Figure 5A).

**Table 1:** Parameters of interest

Parameters of interest	Symbol	Units
Wavelength	λ	nm
Optical power density	OPd	mW/cm^2^
Energy density	E	Joules
Skin tone	–	Melanin μg/mL

### Biphasic dose response in photobiomodulation therapy

The biphasic dose response, also referred to as hormesis, describes a fundamental nonlinear relationship in biological systems: low doses of a stimulus can activate or promote cellular function, whereas higher doses may exert inhibitory or even cytotoxic effects. In the context of tPBM, this phenomenon has been extensively reported and aligns with the Arndt-Schulz law,^13^ typically manifesting as an inverted U-shaped curve. Optimal therapeutic outcomes are observed only within a defined “therapeutic window” previous studies have pointed out that the most commonly used energy densities for treating neurological conditions are between 10 and 30 J/cm^2^.^100^ For example, in cultured cortical neurons, an irradiance of 25 mW/cm^2^ combined with an energy density of 3 J/cm^2^ produced maximal ATP synthesis. In contrast, lower energy densities (0.03 and 0.3 J/cm^2^) and relatively higher energy densities (10 J/cm^2^) yielded only marginal effects, while 30 J/cm^2^ energy density resulted in mitochondrial damage and suppression of bioenergetic activity.^101^ Similarly, Nie et al. reviewed several tPBM studies focused on anti-inflammatory effects, concluding that therapeutic efficacy is highly dose-dependent, with both insufficient and excessive doses diminishing outcomes.^102^ Golovynska further demonstrated dose-dependent effects of red and NIR light on macrophage phagocytosis, cytokine secretion, and mitochondrial function.^103^ Walski et al. also found that low-dose tPBM enhances antioxidant defenses in red blood cell models, whereas high doses increase susceptibility to oxidative stress—even in enucleated cells—highlighting the presence of a comparable therapeutic window.^104^

Moreover, this biphasic response shows strong wavelength specificity. For instance, 810 nm light achieves peak biological effects at an energy density of 3 J/cm^2^, while 980 nm reaches its maximum efficacy at much lower energy densities (0.03 or 0.3 J/cm^2^).^105^ These findings emphasize the critical need to fine-tune dosing parameters for each specific wavelength. The canonical inverted-U response applies across multiple cellular endpoints (mitochondrial activity, ROS/redox balance, inflammatory signaling, and vascular/hemoglobin responses), but the location of the peak on this curve is not a universal constant—rather, it is a function of multiple experimental and subject-specific parameters.^98^ Further investigations into the interplay between wavelength and dosage will be essential for optimizing clinical applications of tPBM in IS (Figure 5B).

### Reconciling dose conflicts via photon fluence and einstein standardization

Reports on optimal dosing in tPBM studies often yield contradictory results, primarily due to discrepancies between reported surface fluence and actual intracranial energy deposition. Conventional energy density (J/cm^2^) fails to calibrate these differences, as it overlooks wavelength-specific photon energy variations and tissue penetration-induced attenuation. Factors such as wavelength, OPd, skull thickness, and skin melanin content substantially influence cortical energy distribution; Monte Carlo simulations demonstrate that, even with identical surface parameters, intracranial doses can vary markedly under different conditions.^106^

To address this challenge, an emerging dosimetry framework proposes photon fluence as a foundational metric.^107^ Photon fluence incorporates per-photon energy (eV) into calculations, enabling the precise quantification of the photon load at the target site. For instance, a 3 J/cm^2^ energy density delivered by 660 nm light (1.9 eV per photon) yields ∼5.7 p.J/cm^2^, substantially higher than ∼4.5 p.J/cm^2^ from 810 nm (1.5 eV). Thus, many apparent contradictions in “low-dose efficacy” across studies likely reflect differences in photon counts rather than fundamental biological variances. For instance, Buzza et al., using the PhoTOS method and MMCLab model for dosimetry simulation, found that 633 nm light requires lower irradiance than 808–830 nm light to achieve equivalent intracranial energy deposition.^108^ Furthermore, to facilitate cross-study comparability, the framework introduces einsteins (ℯ) as a standardized unit.^107^ One ℯ is defined as the photon fluence from 810 nm light at 3 J/cm^2^ (∼4.5 p.J/cm^2^)—chosen for its moderate energy, strong tissue penetration (low water absorption), and widespread use in tPBM literature.^109^ This benchmark allows the conversion of any dose combination into comparable ℯ values; e.g., the aforementioned 3 J/cm^2^ energy density at 660 nm ≈ 1.27 ℯ, versus 0.87 ℯ at 940 nm. Adopting ℯ decouples dose-response comparisons from wavelength dependency, substantially reducing ambiguity in reported efficacies (Figure 5C).

## Latest advances in transcranial photobiomodulation treatment for ischemic stroke

### Animal trials

#### Therapeutic window

Animal studies demonstrate that the therapeutic window for tPBM critically influences its efficacy in IS treatment (Table 2). Various studies, as summarized in the table, applied tPBM at different post-stroke time points, yielding distinct outcomes. For example, Gerace et al.^113^ reported that irradiation (808/905 nm, 7.42 J/cm^2^ energy density) within 30 min post OGD significantly reduced neuronal death in the hippocampal CA1 region and upregulated Bcl-2, an anti-apoptotic protein, whereas delaying to 60 min weakened this effect. Similarly, Strubakos et al.^110^ found that 750/950 nm LED irradiation 90 min post-stroke reduced infarct volume, with 4-h sessions outperforming 2-h ones. These results suggest that tPBM in the acute phase (within hours post-stroke) optimizes cerebral blood flow and curbs inflammation. Conversely, Yokomizo et al.^114^ noted cognitive improvements with irradiation initiated on day 3 post-stroke, indicating potential for neural repair in the later acute phase. Early work by Oron et al.^118^ also showed improved neurological scores with tPBM applied within 24 h, suggesting the therapeutic window varies with the goal—acute protection or long-term repair. Further studies are needed to pinpoint the optimal timing for clinical application.

**Table 2:** Summary of transcranial photobiomodulation animal trials on stroke in the past five years

Author	Animal model	Light parameters	Stroke onset	Irradiation site	Duration	Assessment tools	Results
Strubakos et al., 2020, USA^110^	Sprague-Dawley rats, 90-min transient right MCAO (*n* ≈ 15)	LED 750 and 950 nm (combined), power density 200 mW/cm^2^	90 min post-stroke	Scalp surface	Single session: 120 min or 240 min	MRI to observe infarct volume, CBF, etc.	• Reduced infarct volume. • Decreased ratio of infarct to ischemic risk area. • 4-h irradiation was more effective.
Vogel et al., 2021, Brazil^111^	Wistar rats, PT (*n* = 20)	Diode laser 780 nm; power density 10 mW/cm^2^; energy density 10 J/cm^2^; (reported intensity 0.083 W/cm^2^)	24 h post-stroke	Exposed Skull: 1 mm posterior and 1 mm lateral to bregma	2 min/session, 3x/week for 60 days	EEG recording and omega-3 (Ω-3) after 60 days of treatment, etc.	• Reduced duration of epileptic seizures. • Decreased seizure peak counts in cortices and thalamus. • Decreased epileptiform discharges.
Vogel et al., 2021, Brazil^112^	Wistar rats, PT (*n* = 50)	Diode laser 780 nm; power density 10 mW/cm^2^; energy density 10 J/cm^2^	24 h post-stroke	Exposed Skull: 1 mm posterior and 1 mm lateral to bregma	2 min/session, 3x/week for 60 days	Ischemic volume, Iba1, NeuN, TNF-α, IL-1β, IL-6, IL-10, TGF-β, etc.	• Decreased pro-inflammatory cytokines (TNF-α, IL-1β, IL-6). • Reduced microglial activation. • Decreased infarct area.
Gerace et al., 2021, Italy^113^	Wistar rat pups (*ex vivo* slice model)	NIR laser 808 and 905 nm; spot 3 cm^2^; power density 620 mW/cm^2^; energy density 7.42 J/cm^2^	30 or 60 min after OGD (*ex vivo*)	*ex vivo* hippocampal CA1 slice — direct CA1 irradiation	Immediately after OGD; measured at 0, 30, 60 min	CA1 neurons, NOS, COX-2, p65 NF-κB, Bcl-2, NeuN, GFAP, Iba1, HDN Neuron Density, etc.	• Irradiation within 30 min post-OGD reduced CA1 neuron death (most effective at 7.42 J/cm^2^). • Increased anti-apoptotic protein Bcl-2. • Increased proportion of activated microglia (Iba1^+^).
Yokomizo et al., 2021, USA^114^	Sprague-Dawley rats, GCI (*n* = 95)	Diode laser 808 nm; power density 20 mW/cm^2^; energy density: cortex 2.4 J/cm^2^, hippocampus 0.8 J/cm^2^	Started day 3 after GCI	Scalp: ∼3 mm posterior to the eyes; ∼2 mm anterior to the ears (target area)	2 min/day for 5 days	MWM, BrdU, DCX, NeuN, GFAP, NLRP3, Cle-IL-1β, Iba1, Organelle structures of GFAP and Iba1, etc.	• Improved cognitive function. • Increased NeuN^+^ and DCX^+^ cells in hippocampus. • Decreased astrocyte over-activation and NLRP3 inflammasome.
Kim et al., 2022, Korea^45^	C57BL/6 mice, PT andand MCAO	Implanted LED: 630, 850, 940 nm; power density 17 mW/cm^2^	Pre-stroke (3 days) and post-stroke (4 h)	Directly on cortex (implanted LED array)	Pre-stroke: 20 min, 2x/day for 3 days. Post-stroke: 20 min, 1x/day for 7 days.	Infarct volume, neurological score, NeuN, CD31, Iba1, AIM2, caspase 1, GSDMD, CD86, CD206, IL-1β, IL-10, etc.	• 630 nm LED post-stroke improved chronic phase memory. • Reduced AIM2 inflammasome-mediated neuroinflammation (on the 28th day).
Shalaby et al., 2023, Korea^115^	C57BL/6 mice, PT (*n* = 73)	Diode laser 808 nm; beam diameter 4.17 ± 0.07 mm; power density 325 mW/cm^2^; energy density 40 J/cm^2^	24 h post-stroke	Scalp, olfactory bulb area (between eyelids)	2 min/day for 6 days (Day 2–7)	GFAP, Iba1 (alloantigen inflammatory factor 1), CD31, etc.	• Improved olfaction. • Regulated GFAP andand Iba1 for repair. • Increased anti-inflammatory sICAM-1 and vascular factor CD31.
Feng et al., 2023, USA^54^	Sprague-Dawley rats, PT (*n* = 18)	Diode laser 808 nm, spot diameter 6 mm, power density 350 mW/cm^2^	24 h post-stroke	Skull, 1.8 mm anterior to bregma, 2.5 mm lateral to midline	2 min/day for 7 days	Synaptophysin, MAP2, GFAP and C3d, OGD3, Bcl-xL, BAX, N2a, Spinophilin, Synaptophysin, etc.	• Decreased neurotoxic astrocyte marker (C3d). • Reduced dendritic and synaptic damage. • Increased synaptic integrity markers.
Yokomizo et al., 2024, USA^8^	C57BL/6J mice (Wild-Type and eNOS knockout), MCAO	NIR-II laser 1064 nm, spot diameter 8 mm, power density 50 mW/cm^2^	Pre-treatment: 4 days before stroke	Scalp, top of the skull	5 min/day for 4 days	Infarct volume, CBF, eNOS phosphorylation, NO bioavailability, Evaluation of neurological deficit, etc.	• Pre-treatment reduced infarct volume and neurological deficits. • Increased eNOS phosphorylation, NO bioavailability, and CBF. • Effect was dependent on the eNOS S1176 site.
Feng et al., 2024, USA^116^	Sprague-Dawley rats, PT	Diode laser 808 nm; spot 1.5 cm^2^; power density 350 mW/cm^2^	24 h post-stroke	Skull, 1.8 mm anterior to bregma, 2.5 mm lateral to midline	2 min/day for 7 days	Endostatin level, aging marker (P21) and vascular endothelial marker (CD31), *p*-eNOS, eNOS phosphorylation change, SA-β-gal, γH2AX, etc.	• Increased eNOS phosphorylation. • Decreased endostatin and senescence marker (P21). • Reduced OGD-induced DNA damage.
Feng et al., 2024, USA^117^	Sprague-Dawley rats, PT (n = 6–10/group)	Diode laser 808 nm; spot 1.5 cm^2^; power density 350 mW/cm^2^	24 h post-stroke	Skull, 1.8 mm anterior to bregma, 2.5 mm lateral to midline	2 min/day for 7 days	CBF, BBB permeability, ZO-1, Claudin 5, Testosterone, etc.	• Decreased infarct volume. • Improved glymphatic clearance (increased CSF inflow, ISF clearance). • Reduced neuroinflammation (decreased IL-1β, GFAP/Iba1 activation) and apoptosis.
Kim et al., 2025, Korea^7^	C57BL/6 mice, MCAO (*n* ≈ 36)	Implanted LED 850 nm; power density 89 mW/cm^2^	48 h post-stroke	Skull, 0.5 mm anterior to bregma, 2 mm lateral	10 min/day for 5 days	Infarct volume, NSS, NeuN, CD31, GFAP, Iba1, AQP4, IL-1β, caspase-3, MWM, Y maze, Alexa 647-BSA, etc.	• Decreased infarct volume • Improved glymphatic clearance (increased CSF inflow and ISF clearance) • Increased the number of viable neurons and endothelial cells • Reduced neuroinflammation (decreased IL-1β, GFAP/Iba1 activation) and cell apoptosis.

Abbreviations: 17β-HSD5: 17β-hydroxysteroid dehydrogenase type 5; AIM2: Absent in melanoma 2; AQP4: Aquaporin 4; BBB: Blood-brain barrier; BAX: Bcl-2-associated X protein; Bcl-2: B-cell lymphoma 2; Bcl-xL: B-cell lymphoma-extra large; BrdU: Bromodeoxyuridine; C3d: Complement component 3days; caspase-3: Cysteine-aspartic protease 3; CBF: Cerebral blood flow; CD31: Cluster of differentiation 31 (also known as PECAM-1); CD86: Cluster of differentiation 86; CD206: Cluster of differentiation 206; Cle-IL-1β: Cleaved interleukin-1β; COX-2: Cyclooxygenase – 2; CSF: Cerebrospinal fluid; DCX: Doublecortin; EEG: Electroencephalography; eNOS: Endothelial nitric oxide synthase; GCI: Global cerebral ischemia; GFAP: Glial fibrillary acidic protein; GSDMD: Gasdermin-D; γH2AX: Phosphorylated H2AX histone; HDN: High density nucleus; Iba1: Ionized calcium-binding adapter molecule 1; IL-1β: Interleukin-1β; IL-6: Interleukin-6; ISF: Interstitial fluid; LED: Light-emitting diode; MAP2: Microtubule-associated protein 2; MCAO: Middle cerebral artery occlusion; MRI: Magnetic resonance imaging; MWM: Morris water maze; NeuN: Neuronal nuclei; NFκB: Nuclear factor kappa – B; NIR: Near-infrared; NIR-II: Second near-infrared window; NLRP3: NACHT, LRR and PYD domains-containing protein 3; NO: Nitric oxide; NOS: Nitric oxide synthase; NSS: Neurological severity score; OGD: Oxygen-glucose deprivation; PT: Photothrombosis; ROM: Range of motion; SA-β-gal: Senescence-associated β – galactosidase; sICAM-1: Soluble intercellular adhesion molecule-1; SYP: Synaptophysin; TGF-β: Transforming growth factor-β; TNF-α: Tumor necrosis factor-α; tPBM: Transcranial photobiomodulation; ZO-1: Zonula occludens 1.

#### Irradiation site

The irradiation site directly affects tPBM’s therapeutic efficacy (Table 2). Animal studies, per the table, often target the scalp surface or specific brain regions, such as near the bregma. Yokomizo et al.^8^ used a 1064 nm NIR-II laser on the cranial vertex, significantly boosting CBF and NO bioavailability, implying enhanced outcomes near vascular-rich zones. Likewise, Feng et al.^54^^,^^116^ irradiated anterior sites (1.8 mm anterior, 2.5 mm lateral to bregma) with an 808 nm laser, improving synaptic integrity and cerebrovascular function. Kim et al.^7^^,^^45^ confirmed the efficacy of targeting the sensorimotor cortex via implanted LEDs. Early studies, such as Lapchak et al.,^119^ showed that irradiating the cortex or deeper regions reduced infarct volume, though limited penetration depth may restrict efficacy. Differences in outcomes likely arise from variations in light penetration, tissue scattering, and the functional role of targeted areas, warranting refined irradiation strategies.

#### Treatment frequency

Treatment frequency markedly influences tPBM outcomes (Table 2). The table lists protocols ranging from daily sessions (e.g., Kim et al.,^7^ 10 min/day for 5 days) to thrice-weekly applications (e.g., Vogel et al.,^112^ 2 min/session for 60 days). Multiple sessions typically enhance neural repair; Yokomizo et al.^114^ reported elevated hippocampal NeuN and DCX expression and better cognition after 5 consecutive days of irradiation. In contrast, Strubakos et al.^110^ found that a single 4-h session reduced infarct volume, though repeated treatments may offer superior long-term benefits. Early research by Uozumi et al.^58^ indicated that multi-day irradiation outperforms single sessions in promoting CBF and neurogenesis. Repeated applications likely amplify mitochondrial activity and neurotrophic factor expression, but frequency must be optimized to avoid thermal tissue damage.

#### Laser vs. light emitting diode

The choice of light source is a pivotal factor in tPBM studies (Table 2). The table shows that both lasers (e.g., 808 nm, Yokomizo et al.^8^) and LEDs (e.g., 630 nm, Kim et al.^45^) yield significant benefits. Lasers, with greater coherence and penetration, excel in specific contexts; Feng et al.^117^ used a 350 mW/cm^2^ 808 nm laser to lower endostatin levels and stimulate angiogenesis. However, LEDs (e.g., 850 nm, Kim et al.^7^) are increasingly preferred for their cost-effectiveness, safety, and broader coverage. Strubakos et al.^110^ observed reduced infarct volume with 750/950 nm LEDs and no adverse effects. Early insights from Hamblin^70^ highlight lasers’ superiority in deep tissue penetration, while LEDs offer safer, prolonged use. Comparative studies in specific stroke models are essential to standardize protocols.

### Human studies

#### Positive signals from small-scale clinical studies

Preliminary evidence from several small-scale clinical studies suggests that tPBM holds considerable clinical potential in the management of IS. When utilized as an adjunctive therapy with conventional rehabilitation, tPBM has been shown to effectively improve specific neurological deficits in patients (Table 3). For example, Naeser et al.^120^ applied 633/870 nm LEDs three times weekly for six weeks in six patients with IS with aphasia, significantly enhancing naming ability, particularly when targeting DMN nodes. Estrada-Rojas et al.^124^ combined LED helmet irradiation with speech therapy, markedly improving articulation and expressive language in a patient five months post-stroke. Paolillo et al.^123^ used a diode laser alongside neuromuscular electrical stimulation, boosting cognition and quality of life in patients with chronic hemiplegic. Ashrafi et al.^121^ integrated a diode laser with low-frequency electromagnetic fields, significantly improving neurological function (NIHSS) and cognitive performance (MMSE).

**Table 3:** Summary of transcranial photobiomodulation human studies on stroke in the past five years

Author	Study design	Participants, Age, Number	Light parameters	Stroke onset	Irradiation site	Duration	Assessment tools	Results	Serious adverse events
Naeser et al., 2020, USA^120^	Case series	Post-stroke aphasia *n* = 6 (male) Age: 46–69 (average 58.7 years old)	LED, 633 nm + 870 nm; power density: 22.2 mW/cm^2^, 500 mW; energy density: (1) 13 J/cm^2^; (2) 39 J/cm^2^; (3) 13 J/cm^2^; (4) 26 J/cm^2^	2-18 years	Four sites: (1) Bilateral (left/right hemispheres) + midline (including bilateral SMA); (2) Ipsilateral left brain (lesion side); (3) Ipsilateral left brain +1 midline node of the DMN (mPFC); (4) Ipsilateral left brain +2 midline nodes of the DMN (mPFC + precuneus)	Frequency: 3 times/week Total: 6 weeks (18 sessions) Session: 19.5–39 min	BDAE, BNT, PNT, FAS, etc.	• Confirmed that NIR light can act on the underlying cerebral cortex. • Protocols targeting the ipsilateral brain (2) and DMN nodes (4) effectively improved naming ability. • Protocol (4) also significantly enhanced the functional connectivity (FC) of the DMN, SN, and CEN.	None reported. Safe even in patient with epilepsy.
Ashrafi et al., 2020, Iran^121^	Single-blind randomized controlled trial	Acute IS *n* = 52 Age (Exp. group): 63.5 ± 14.3 years Age (Ctrl. group): 65.9 ± 15.7 years	Diode laser 840 nm, 50 Hz; power density 12 mW/cm^2^; combined with 1 mT, 50 Hz extremely low-frequency electromagnetic field	First onset, single hemisphere, symptoms ≤48 h	12 scalp positions based on the 10–20 EEG system	Frequency: Daily Total: 5 days Session: 45 min	NIHSS, BI, mRS, GDS, MMSE, etc.	• The combined treatment of tPBM and ELF-EMF improved neurological function, functional status, cognitive ability, and depressive symptoms. • This combination was more effective than standard treatment alone.	None reported. Mild headache (*n* = 1) and warmth (*n* = 3) noted.
Morse et al., 2021, USA^122^	Human anatomical study	Fresh human cadavers *n* = 4 (2 African American females, 1 Caucasian female, 1 Caucasian male)	750 nm + 940 nm; power density (whole head): 2–4 mW/cm^2^; (separated tissues): 0.5–1 mW/cm^2^	–	Cadaver head: frontal, parietal, occipital, temporal lobes; separated tissues: skin, subcutaneous fat, temporalis muscle, skull, cerebral gray matter	Single irradiation	Optical power meter, integrating sphere, digital caliper, etc.	• 750 nm and 940 nm IRL can penetrate 4 cm of human head tissue. • The parietal lobe showed the highest penetration rate. • 940 nm light demonstrated slightly better penetration than 750 nm. • Light transmission was similar across different skin colors. • The skull and cerebral gray matter were the primary attenuators.	None reported; but occasional skin burns from blood stasis were observed, which resolved after repositioning.
Paolillo et al., 2023, Brazil^123^	Mixed single-blind randomized controlled trial and *ex vivo* study	Mainly IS (chronic) *n* = 18 (13 males, 5 females), Age (Exp. group): 59 ± 11 years Age (Ctrl. group): 63 ± 8 years	Diode laser (660 nm, 808 nm, 980 nm); energy 43.2 J/cm^2^; power density 400.8 mW/cm^2^	12 months	All 15 head regions	Frequency: Once a week Total: 12 weeks Session: 15 min	Grip strength, EBES, MAS, VAS, MMSE, FIM, WHOQOL-BREF, etc.	• The combination of tPBM and NMES improved cognition, pain, manual flexibility, and quality of life. • This combination was more effective than sham laser + NMES. • *Ex vivo* experiments showed 808 nm has better skull penetration than 660 nm.	None reported.
Estrada-Rojas et al., 2023, USA^124^	Case report	Post-left-brain IS with aphasia and dysarthria *n* = 1 (female) Age: 38	(1) LED cluster head (630 nm, 660 nm, 850 nm); power density 200 mW/cm^2^, energy 12 J/cm^2^; (2) LED helmet 810 nm; power density 24 mW/cm^2^, energy 28.8 J/cm^2^	5 months	(1) Left hemisphere language network (8 targets including Broca’s area, Wernicke’s area); (2) Whole scalp (both hemispheres)	Frequency: Twice a week Total: 5 months (30 sessions) Session: 45 min (28 min tPBM +17 min speech therapy)	Clinical dysarthria assessment, BDAE, Oral ability assessment, etc.	• Combined tPBM and speech-language therapy was significantly more effective than speech therapy alone in improving dysarthria and expressive language ability.	None reported.

Abbreviations: BDAE: Boston diagnostic aphasia examination; BI: Barthel index; BNT: Boston naming test; CEN: Central executive network; DMN: Default mode network; EBES: Subjective well-being scale; EEG: Electroencephalography; ELF-EMF: Extremely low-frequency electromagnetic field; FAS: Functional activities questionnaire; FC: Functional connectivity; FIM: Functional independence measure; GDS: Geriatric depression scale; IS: Ischemic stroke; IRL: Infrared light; LED: Light-emitting diode; MAS: Modified Ashworth scale; MMSE: Mini-mental state examination; mPFC: Medial prefrontal cortex; mRS: Modified Rankin scale; NIHSS: National Institutes of Health stroke scale; NIR: Near-infrared; NMES: Neuromuscular electrical stimulation; PNT: Picture naming test; SN: Salience network; SMA: Supplementary motor areas; tPBM: Transcranial photobiomodulation; VAS: Visual analogue scale; WHOQOL-BREF: World Health Organization quality of life – BREF.

Beyond cranial applications, direct tPBM on the paretic limbs of patients with stroke has also demonstrated positive outcomes. In a randomized controlled trial, das Neves et al.^125^ found that 808 nm tPBM on the upper limbs of patients with post-stroke spastic hemiplegic significantly improved range of motion (ROM), reduced pain, and enhanced muscle torque and electromyography (EMG) parameters. Similarly, das Neves et al.^126^ demonstrated that long-term 808 nm tPBM reduces spasticity and improves motor function in patients with chronic stroke, as indicated by lower Ashworth scale scores. dos Reis et al.^127^ showed that 808 nm low-level laser irradiation on spastic muscles immediately reduces fatigue and increases grip strength. Recent studies suggest tPBM enhances skeletal muscle mitochondrial function and reduces local inflammation to improve motor performance.^128^ Başaran et al.^129^ propose that tPBM may aid post-stroke limb rehabilitation by modulating the muscle microenvironment, such as lowering pro-inflammatory IL-6. These findings indicate localized tPBM on limbs can complement cranial irradiation, particularly for patients with spasticity and motor deficits post-stroke.

But the potential mechanisms underlying these two distinct application routes may differ. Cranial tPBM is thought to exert its effects primarily through “direct photoneuromodulation” whereby photons penetrate the skull to directly enhance mitochondrial function and reduce oxidative stress within the brain tissue. In contrast, the mechanism for peripheral limb tPBM appears to be more reliant on the multifaceted modulation of the “local muscle microenvironment.”^128^ This includes improving the metabolic state of the muscle (e.g., by reducing blood lactate),^130^ exerting analgesic effects (e.g., by modulating prostaglandins or promoting endorphin release),^131^ and facilitating tissue remodeling (e.g., by improving local vascularization and increasing angiogenic factors).^132^ Together, these mechanisms help to alleviate the structural and functional changes in muscle fibers caused by spasticity, thereby improving joint mobility and overall function.

#### Challenges revealed by large-scale trials

In contrast, the results from large-scale, acute-phase clinical trials—specifically the NeuroThera Efficacy and Safety Trials (NESTs)—were disappointing. The initial findings from NEST-1 (*n* = 120; ages 40–85) were promising: a single 808 nm laser application to the whole scalp within 18 h of stroke onset (energy density of 1.2 J/cm^2^) resulted in 70% of patients in the active treatment group achieving a successful outcome (defined as a ≥9-point decrease in NIHSS score) at 90 days, a rate significantly higher than the 51% observed in the sham group (*p* < 0.05),^133^ However, the subsequent NEST-2 trial (*n* = 660; ages 40–90) only demonstrated a non-significant positive trend (36.3% vs. 30.9% favorable outcome),^134^ Ultimately, the NEST-3 trial, which was planned for 1000 patients, was terminated early after the Data and Safety Monitoring Board concluded that it was unlikely to show a neuroprotective effect.^135^The trajectory of the NEST series from initial optimism to eventual failure does not negate the intrinsic therapeutic potential of tPBM. Instead, it critically highlights the complex challenges involved in translating this technology from laboratory and animal models to successful clinical application in complex human diseases. The reasons for this failure can be attributed to several key factors:

First, inadequate light penetration due to anatomical differences. The human skull is considerably thicker than that of laboratory animals.^136^ Based on the parameters used in the NEST trials (10 mW/cm^2^ irradiance, 1.2 J/cm^2^ energy density), it has been estimated that after penetrating the average 7–10 mm thick skull and an additional 1–2 mm of extracerebral tissue, the maximum penetration depth into the cerebral cortex would be only 8 mm.^137^ Crucially, the NEST trials did not exclude patients with deep ischemic lesions. Consequently, after significant attenuation and scattering by the scalp, cranium, and gray matter, the effective light dose was likely insufficient to reach the ischemic penumbra. Second, non-specific targeting and heterogeneous dose distribution. The NEST trials employed a whole-head irradiation protocol that was not individualized based on lesion location. Research has demonstrated that the absorption of NIR light varies significantly across different cranial regions. One cadaver study, for example, showed that the transmittance of 830 nm light was 0.9% in the temporal region, 2.1% in the frontal region, and as high as 11.7% in the occipital region.^138^ Furthermore, another study confirmed that even within the same region, such as the orbitofrontal cortex, NIR light absorption differed across various emitter channel locations.^139^ This evidence indicates that a uniform surface application leads to a highly heterogeneous intracranial dose distribution. Combined with a limited number of 20 fixed emitter channel locations, this approach may have resulted in energy dispersion, preventing the formation of a therapeutic dose in critical areas adjacent to the penumbra. Third, the treatment protocol parameters may have been suboptimal. The low-power, continuous wave (CW) mode used in the trials may be less efficient for deep-tissue energy delivery. Substantial evidence suggests that a pulsed wave (PW) mode offers advantages, as it can deliver higher peak power to deeper tissues without causing superficial thermal damage,^140^ thereby more effectively stimulating mitochondrial function within the ischemic penumbra.^141^ Moreover, the NEST dose (808 nm, 1.2 J/cm^2^ energy density), when converted to a standardized photon fluence unit, is equivalent to only 0.4 ℯ.^107^ This dose was likely too low to both overcome cranial attenuation and activate the neuroprotective mechanisms, such as mitochondrial ATP synthesis, required to salvage penumbral tissue. Finally, given the complex and sustained pathophysiological cascade following a stroke, a single, 2-min tPBM intervention was likely insufficient in both intensity and duration to meet the continuous demand for cellular repair.

#### Future perspectives and recommendations for preclinical and clinical study designs

Synthesizing the clinical evidence presented above, future preclinical and clinical research must be guided by the core principles of precision, individualization, and reproducibility. This approach will provide a roadmap for transitioning tPBM from a promising intervention into an evidence-based therapeutic modality.

First and foremost, dosimetric standardization is required. Future studies should be committed to establishing a standardized dosimetry system centered on intracranial photon fluence and should report more comparable, standardized units such as the einstein (ℯ) to enable meaningful cross-study comparisons. Critically, computational modeling should be employed prior to clinical study design to estimate and optimize the photon fluence that can actually reach the ischemic penumbra, thereby accounting for individual variables such as skull thickness, skin tone, and even age.^142^ Second, drawing lessons from the NEST trials, the research focus should shift from “broad-field irradiation” to more precise, “individualized targeting” strategies. For instance, guided by functional imaging data (e.g., fMRI), treatments could be focused on specific, functionally relevant brain networks that are impaired but retain plasticity, such as the successful targeting of DMN nodes in the study by Naeser et al.^120^ Concurrently, study designs should incorporate patient stratification based on lesion location (e.g., considering the exclusion of patients with deep subcortical lesions or designing specific, higher-energy protocols for them) to ensure therapeutic efficacy. Furthermore, it is essential to explore phase-specific treatment protocols tailored to the distinct pathophysiological stages of IS. Based on the “biological resonance” hypothesis,^143^ a PW mode with a higher frequency (e.g., 40 Hz) may be more advantageous during the acute phase. This frequency aligns with the gamma rhythm associated with higher cognitive functions, and since driving gamma oscillations has been shown to modulate neuroimmune responses,^144^ a 40 Hz PW might more effectively activate the protective functions of microglia to achieve maximal neuroprotection. In contrast, during the chronic phase, a lower frequency (e.g., 8–12 Hz) that approximates the alpha rhythm could be explored. Given that tPBM has been demonstrated to modulate alpha activity in healthy subjects^145^—a rhythm associated with enhanced memory and a state of relaxed alertness—it may help to stabilize mood and promote cognitive FC network remodeling in patients with IS. Finally, the success of the vast majority of small- and medium-sized studies has often stemmed from combination therapy. Therefore, investigating the synergistic effects of tPBM with other IS treatment modalities (such as conventional rehabilitation, physical therapy, or other neuromodulation techniques) and with peripheral limb tPBM represents a critical direction for future research. This multimodal approach may hold greater potential for clinical translation than any standalone therapy has previously demonstrated.

## Systemic effects

Emerging evidence indicates that tPBM’s therapeutic effects extend beyond directly irradiated brain tissue, with remote irradiation potentially yielding systemic benefits via immune and circulatory pathways. Studies suggest intravascular laser irradiation (ILIB) or laser application to non-cranial sites, such as the back or limbs, can enhance systemic circulation and the biological activity of blood components, indirectly improving brain function.^146^ For instance, Choi et al.^147^ observed increased peritoneal leukocytes, reduced microglial activation, smaller infarct volumes, and better neurological function in MCAO model rats after three weeks of 710 nm LED irradiation above their cages. Li et al.^148^ reported significant muscle strength gains, reduced serum homocysteine, and increased CBF via SPECT in a subacute IS patient with left-sided motor deficits after three ILIB courses, despite no direct cranial irradiation. These results suggest that peripheral irradiation can offer neuroprotection by modulating the immune environment and microinflammation, even without brain targeting. Such systemic effects may involve tPBM-induced platelet mitochondrial ATP production and stem cell migration to damaged brain regions.^48^ Additionally, a whole-body LED light cabin (660/850 nm) has shown potential to enhance muscle performance and reduce fatigue, suggesting its use as an adjunctive stroke rehabilitation tool.^149^ Some studies propose remote tPBM may influence the gut-brain axis and immune cell migration, providing neuroprotection.^150^ However, a meta-analysis by Álvarez-Martínez et al.^151^ found no significant effect of systemic tPBM on exercise performance or recovery in healthy adults, suggesting its efficacy may be pathology-specific, possibly more evident in ischemic or neurologically impaired states. Further research is needed to clarify the mechanisms, such as immune cell and cytokine dynamics, to expand tPBM’s therapeutic scope.

## Safety profile

The safety of tPBM in the treatment of IS has been preliminarily established across various studies. Animal models have consistently reported no serious adverse events (SAEs), and early human clinical trials have similarly indicated good tolerability. For instance, Naeser et al.^120^ found no seizure induction with 633/870 nm LED therapy, even in a patient with a history of epilepsy. Both Paolillo et al.^123^ and Estrada-Rojas et al.^124^ reported no SAEs, while Ashrafi et al.^121^ documented only one mild headache and three cases of slight warmth, none requiring intervention. A human safety study showed that even at a high flux of 264 J/cm^2^ energy density using an 830 nm panel, tPBM did not induce DNA damage.^152^ A systematic review suggests that although tPBM’s clinical efficacy remains modest, repeated sessions can yield moderate benefits, and its strong safety profile positions it as a promising adjunctive therapy. Current LED-based tPBM devices are cost-effective and simple, with potential for home-use development to improve adherence.^153^ These findings are consistent with the latest 2025 evidence-based consensus, which regards PBM as a safe treatment modality and specifically notes that red light PBM does not induce DNA damage. This consensus also affirms the safety of PBM for improving cognition and for peripheral nervous system conditions, further supporting its neural safety in cranial applications.

However, despite this strong safety record, a balanced perspective requires a critical evaluation of the limitations of current evidence, particularly concerning the potential risks of long-term application. First, large-scale, long-term safety data for chronic, repeated tPBM applications are currently scarce—a point echoed by the 2025 consensus,^154^ which explicitly states, “Data on the long-term effects of prolonged use of photobiomodulation are limited.” While the side effects of single sessions are typically mild and self-resolving, the potential for cumulative thermal effects from prolonged or high-frequency tPBM has not yet been adequately determined. This is a critical question for patients with chronic stroke who may require lifelong rehabilitation management. Second, the safety of tPBM in patients with IS with multiple clinical comorbidities needs further investigation. Patients with IS frequently present with comorbidities such as diabetes, dementia, or a history of photosensitivity, and the 2025 consensus advises caution when using tPBM in patients with a history of photosensitivities.^154^ It remains unknown whether these comorbidities might affect a patient’s tissue response and safety threshold for tPBM. Finally, the individualized adjustment of treatment parameters is crucial for ensuring safety; for instance, Chao et al.^80^ noted mild headaches linked to a 40 Hz pulse frequency, which resolved after switching to 10 Hz, highlighting the importance of tailored parameter adjustments, and the consensus advises that patients with darker skin phototypes may face a slightly elevated risk of side effects, suggesting that more conservative parameters should be employed. Furthermore, clinicians should opt for certified devices, as some market products lack medical approval. In conclusion, future personalized protocols should integrate imaging-based monitoring and dose-escalation strategies to establish an optimal balance between long-term safety and therapeutic efficacy.

## Future perspectives

tPBM, which delivers NIR light non-invasively to deep brain tissues, holds significant promise for IS treatment but requires further research to address existing challenges. Studies confirm tPBM’s ability to enhance neurological function and reduce infarct size in animal models and select human trials, yet clinical evidence remains limited, and standardized protocols are absent. A key challenge is identifying optimal irradiation sites, as different cortical regions may engage distinct neural networks.^81^ Future studies should refine site selection—comparing motor cortex, language areas, or prefrontal cortex stimulation—to guide personalized interventions. Optimizing parameters such as wavelength, power density, duration, and pulse mode is equally critical. NIR II (1000–1700 nm) light, with deeper penetration, appears more promising than NIR-I (630–900 nm) light,^155^ but robust randomized controlled trials (RCTs) are needed for confirmation. The mechanisms by which tPBM protects the BBB remain underexplored. Kim et al.^7^ showed that 850 nm light boosts CSF clearance and preserves BBB integrity, while Ting et al.^156^ found 808 nm laser increases BBB permeability in cellular models, possibly via metalloproteinase activity and tight junction protein changes. These findings suggest tPBM’s dual role in BBB regulation, varying with parameters such as wavelength or intensity. Future research should investigate how to tailor tPBM to either safeguard the BBB or enhance drug delivery to the central nervous system, offering new therapeutic possibilities. Exploring tPBM’s impact on neurovascular unit integration—particularly through neuron-glia-vessel signaling to stabilize the brain microenvironment—could further advance IS rehabilitation and neuroprotection. Additionally, the synergy of tPBM with multimodal approaches, such as stem cell therapy or rehabilitation training, merits investigation. Preliminary evidence from Estrada-Rojas et al.^124^ suggests that combined treatments may enhance recovery. However, widespread clinical adoption depends on large-scale, standardized RCTs. Technologically, developing portable LED devices with personalized adjustments based on scalp-to-brain distance could propel tPBM toward precision IS rehabilitation, addressing limitations seen in the NEST-3 trial.

## Data and code availability

Data sharing is not applicable to this article as no new data were created or analyzed in this study. The pictures in this article were Created in https://BioRender.com.

## Acknowledgments

This work was supported by the Ningbo Rehabilitation Hospital hospital level key project (Project Number: 2024KY01), Ningbo Rehabilitation Hospital hospital project (Project Number: 2022KY05), Project of Ningbo Leading Medical Health Discipline (Project Number: 2022-G02), the 2023 Annual Pathophysiology Technology Research Key Laboratory Open Fund of 10.13039/100017710Zhejiang Province (Project Number: 202307), 10.13039/501100015378Medicine and Health Care in Zhejiang Province Science and Technology Plan Projects of Traditional Chinese Medicine (Project Number: 2024ZL954), and the 10.13039/100017330Collaborative Innovation Center for Health and Elderly Care Application Technology and Standards (Project Number: JKYL2022010).

## Author contributions

L.T. and X.L. designed and wrote the article. J.L. and Y.L. revised the article. Y.W. and M.T. gave constructive advice and participated in proofreading this article. All authors contributed to the article and approved the submitted version.

## Declaration of interests

The authors declare that they have no known competing financial or non-financial interests that could be perceived to influence the results or interpretation of the work reported in this article. No potential conflict of interest was reported by the author(s).
